# Interaction of 8-Hydroxyquinoline with Soil Environment Mediates Its Ecological Function

**DOI:** 10.1371/journal.pone.0012852

**Published:** 2010-09-21

**Authors:** Inderjit, Devika Bajpai, M. S. Rajeswari

**Affiliations:** 1 Department of Environmental Biology, University of Delhi, Delhi, India; 2 Centre for Environmental Management of Degraded Ecosystems (CEMDE), University of Delhi, Delhi, India; 3 Department of Botany, Acharya Narendra Dev College, University of Delhi, Delhi, India; University of Kansas, United States of America

## Abstract

**Background:**

Allelopathic functions of plant-released chemicals are often studied through growth bioassays assuming that these chemicals will directly impact plant growth. This overlooks the role of soil factors in mediating allelopathic activities of chemicals, particularly non-volatiles. Here we examined the allelopathic potential of 8-hydroxyquinoline (HQ), a chemical reported to be exuded from the roots of *Centaurea diffusa*.

**Methodology/Principal Findings:**

Growth bioassays and HQ recovery experiments were performed in HQ-treated soils (non-sterile, sterile, organic matter-enriched and glucose-amended) and untreated control soil. Root growth of either *Brassica campestris* or *Phalaris minor* was not affected in HQ-treated non-sterile soil. Soil modifications (organic matter and glucose amendments) could not enhance the recovery of HQ in soil, which further supports the observation that HQ is not likely to be an allelopathic compound. Hydroxyquinoline-treated soil had lower values for the CO_2_ release compared to untreated non-sterile soil. Soil sterilization significantly influenced the organic matter content, PO_4_-P and total organic nitrogen levels.

**Conclusion/Significance:**

Here, we concluded that evaluation of the effect of a chemical on plant growth is not enough in evaluating the ecological role of a chemical in plant-plant interactions. Interaction of the chemical with soil factors largely determines the impact of HQ on plant growth.

## Introduction

Plant-released chemicals can suppress the growth of other plants, a process known as allelopathy. One of the reasons for recent controversies in allelopathy [1]–[5] is the use of an over-simplified experimental approach, which ignores the role of soil factors in mediating allelopathic activities. The use of artificial growth medium and extraction procedures [6] neglects the importance of the ecological functions of soils.

Vivanco et al. [6] reported 8-hydroxyquinoline (hereafter referred as HQ) in the root exudates of *C. diffusa* collected in the Murashige and Skoog medium. Root exudates were sterilized at 121°C for 30 min. at 15 lb pressure prior examining phytotoxic activities. While this eliminates the possibility that inhibition is due to microbial effects, biological activity determined with autoclaved root exudates is not sufficient to demonstrate the allelopathic potential of root exudates in natural situations. *In vitro* experiments were carried out to examine the phytotoxicity of HQ. It was claimed that high levels of HQ (264.12±21.2 µg/g soil *c.* 10 cm from taproots of *C. diffusa*) in *C. diffusa-*invaded North American soils justified results of *in vitro* growth bioassays, and direct role of HQ in growth suppression of assay species was argued. However, Vivanco et al. [6] did not consider the ecological functions of soil microbes in *in vitro* growth bioassays. While investigating the impact of herbivory on allelopathic performance of *C. diffusa*, Norton et al. [2] could not detect HQ from greenhouse soils previously used to grow *C. diffusa*-*Artemisia frigida* (no herbivory or with herbivory). Since HQ shows significant affinity for metal ions, the addition of EDTA (disodium ethylene diaminetetraacetate) to the mobile phase of HPLC solvent significantly increased the recovery of HQ, which indicated the presence of HQ-Fe complex. This is an important step in understanding of the ecological role of HQ. These authors did not find any evidence of HQ contributing to the invasion success of *C. diffusa*. Further carrying forward the work on HQ allelopathy, Tharayil et al. [7] found that HQ production was high in Fe-aided *C. diffusa* plants, and concluded that one of probable functions of HQ might be Fe acquisition, especially in calcareous soils where *C. diffusa* invades. It was suggested that HQ is primarily exuded to acquire Fe for the donor plant, and the reduced phytotoxicity of HQ was argued due to the binary mixture of HQ and metal ions. These authors carried out their work in hydroponic solutions mainly because manipulating the availability of micronutrient in alkaline-calareous soils was realistically not possible. This, however, leaves open the question of how soil factors and microorganisms impact the allelopathic activities of HQ. The impact of HQ on soil microbial activity and bacterial community structure is reported [8], but any role of soil microbial activity in manipulating allelopathic potential of HQ has not been examined.

We carried out a study to examine the ecological role of HQ in soil environment by examining (i) growth performance of certain species and HQ recovery in HQ-treated non-sterile or sterile soils, (ii) impact of HQ treatments on microbial activity, and soil properties such as organic matter, phosphate-P and total organic nitrogen, (iii) effect of soil modifications (glucose and organic matter amendments) on growth performance and HQ recovery.

## Materials and Methods

### Growth Bioassays

50 g soil samples (sandy loam; pH, 6.97±0.03; Electrical conductivity, 0.14±0.01 mMhos/cm; organic matter, 3.66±0.29%) were each placed in a Petri dish (9 cm) and irrigated with 15 mL of 266.66, 533.33 and 800 mg HQ/L to obtain 80, 160 or 240 µg HQ/g soil. Soil treated with 15 mL distilled water (hereafter referred as water) is identified as control. Ten seeds of *Brassica campestris* or *Phalaris minor* were placed on control or treated soils. Each treatment was replicated 5 times. Data on root length were collected 7 d after seed germination. The experiment was replicated five times. Average environmental conditions were average day/night temperature of 21/18°C, 12 h photoperiod. Data were analyzed using independent sample t-test.

### Manipulation of 8-Hydroxyquinoline Phytotoxicity

#### Sterile soil

One way of analyzing the role of soil microbes in determining the allelopathic potential of an alleged allelochemical is through making comparative growth studies in non-sterile and sterile soils treated with different amounts of the allelochemicals [4], [5]. Non-sterile soil previously taken for growth experiments was sterilized for 3 successive times for 30 min. at 121°C and at 103 KPa without intervals between sterilization. 50 g sterile soil samples were treated with 15 mL of 266.66, 533.33 and 800 mg HQ/L to obtain 0, 80, 160 or 240 µg HQ/g sterile soil. Our preliminary experiments suggest that 15 mL solution was required to moisten 50 g soil, which did not create stagnated conditions. Ten seeds of *B. campestris* or *P. minor* were placed on control or treated soils. Each treatment was replicated 5 times. Data on root length were collected 7 d after sowing. Average environmental conditions were day/night temperature of 21/18°C, 12 h photoperiod. Data were analyzed using independent sample t-test.

### Organic matter modification

Allelochemicals bind to the surface of soil organic matter that prevents their metal oxidation [9]. We therefore examined the effect of organic matter-enriched soil treated with HQ on seedling growth of *B. campestris* or *P. minor*. The compost was added to experimental soil to obtain organic matter content of 1.5% and this mixture was then treated with HQ to obtain final concentrations of 0, 80, 160 or 240 µg HQ/g soil. 50 g soil mixture was placed in 71.5 cm^3^ pots and treated with 15 mL of 0, 266.7, 533.3 or 800 mg HQ/L to obtain final concentrations of 0, 80, 160 or 240 µg HQ/g soil mixture. Ten seeds of *B. campestris* or *P. minor* were sown in each pot, and irrigated daily with 5–10 mL water. Data on root length were recorded after 7 d. Each experiment was replicated 5 times. Average environmental conditions were day/night temperature of 21/18°C, 12 h photoperiod. Data were analyzed using independent sample t-test.

### Glucose amendment

Many species of soil microbes have a preference to use glucose as a carbon (C) source compared to phenolic compounds [10], [11]. We hypothesized that HQ might exhibit phytotoxicity after glucose amendment because microbes might use glucose as preferred labile C source. To study the impact of glucose amendments on HQ phytotoxicity, solution containing 0, 582 or 1129 µg glucose per mL water were prepared, which corresponds to 0, 233 and 450 µg C/g soil, respectively [12]. 50 g soil samples were was treated with 12 mL of approximate concentrations of HQ and 3 mL of approximate levels of glucose to get final concentrations of 0, 80, 160 and 240 µg HQ/soil with 0, 233 or 450 µg glucose/g soil. Ten seeds of *B. campestris* or *P. minor* were sown in each pot, and irrigated daily with 5–10 mL water. Data on root length were recorded after 7 d. Each experiment was replicated 5 times. Average environmental conditions were day/night temperature of 21/18°C, 12 h photoperiod. Data were analyzed using independent sample t-test.

### Hydroxyquinoline Recovery in Soil

The recovery of HQ was examined in non-sterile, sterile, Organic matter (OM)-enriched and glucose-modified soils when treated with 0, 80, 160 or 240 µg HQ/g soil and incubated at 25°C for 24 h. Soil was not completely dried after 24 h but similar incubation conditions ensure consistent water content at every sampling. 5 g soil was soaked in 10 mL methanol for 24 h followed by filtration through 0.2 µm PES filter media (Whatman, Schleicher and Schuell). Hydroxyquinoline level in methanolic soil extract of each soil was determined by using high-performance liquid chromatography (HPLC) (Waters Corp., Wilfred, U.S.A), employing a Waters Spherisorb 5 µm CNRP column (4.6×250 mm Analytical Column) with a flow- rate of 1 mL/min and UV absorbance at 250 nm. Mobile phase solution A consisted of 0.1% ortho-phosphoric acid in water and Solution B consisted of 100% acetonitrile. The isocratic mobile phase was started with 90% mobile phase A and 10% mobile phase B. 10 µL of sample was injected in the partial loop needle overfill (PLNO) mode at sample vial temperature of 10°C and column temperature of 40°C. The run time for each sample was 15 min, and the retention time of HQ was 6.7–6.87 min. The limit of detection was 0.4 ppm and method quantification limit was 1.4 ppm.

### Soil Respiration

We measured soil CO_2_ release by chemical titration to study the effect of HQ treatments on soil microbial activity. A 5-cm Petri dish containing 10 mL of 0.1 N NaOH was placed in the larger box (size) filled with 250 g soil. Non-sterile soil was then treated with HQ to get a concentration of 0, 80, 160 or 240 µg HQ/g soil. Each box was then covered and sealed to avoid any loss of CO_2_. Soil was incubated for 24 h, and was terminated by adding 1 mL of 0.1 N BaCl_2_ to the NaOH. A 10 mL NaOH was taken from blank, control or treatment and titrated against 0.1 N HCl using phenolphthalein as an indicator. Three replicates were used. The amount of CO_2_ released was calculated following [13].
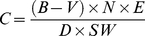



Where

C =  mg of CO_2_ released per gram soil per hour

B =  volume (mL) of acid needed to titrate the NaOH to control to get endpoint

V =  Volume (mL) of acid needed to titrate the NaOH exposed to soil atmosphere to get end point.

N =  Normality of HCl acid (0.1 N)

E =  Equivalent weight of CO_2_ (i.e., 22)

D =  Duration of incubation (h)

SW =  Soil weight (g)

### Soil Chemical Analysis

Soil (non-sterile and sterile) was incubated with 0, 80, 160 or 240 µg HQ/g soil for 24 h and then analyzed for organic matter, exchangeable PO_4_-P and total organic nitrogen. Soil organic matter (SOM) was determined using the chromate titration method [14]. To determine PO_4_-P, 5 g soil was soaked with 25 mL of 2.5% acetic acid, shaken for 30 min followed by filtration. Exchangeable PO_4_-P was estimated using molybdenum blue method (Allen, 1989). Total organic N was determined by digesting 1 g soil using kjeldahl method, and total organic N concentration was determined using indophenol method [15]. All analyses were done using six replicates. Two-way ANOVA was carried out for the effect of soil sterilization and HQ concentration and their interaction on SOM, PO_4_-P and total organic N levels in soil.

5 g of soil was soaked with 25 mL water and shaken for 1 h followed by filtration through Whatman # 1. Soil filtrate was used to measure total phenolics using Folin and Ciocalteu's reagent [16]. Folin reagent is known to be reduced by amino acid and proteins but the reduction by these compounds is nonsignificant compared to reduction by phenolics [17]. In the present study, we determined total phenolics content and not the absolute concentration of phenolics. Folin ciocalteu reagent is used to quantify the variation in the concentration of phenolics [18].

## Results

### Non-Sterile and Sterile Soils

**Figure 1:**
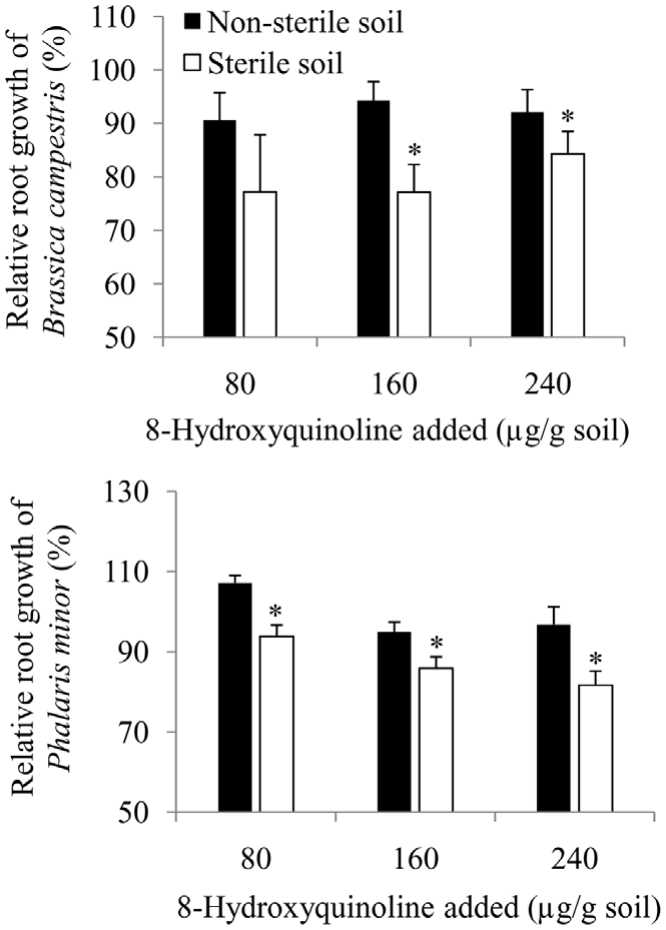
Relative root length (%) of *Brassica campestris* and *Phalaris minor* when grown in non-sterile or sterile soil treated with 0, 80, 160 or 240 µg 8-hydroxyquinoline/g soil. Root length of untreated control (0 µg HQ/g soil) is taken as 100%, and root length of both the assay species were calculated to the zero level of HQ. Bars indicate 1 SE. Asterisks indicate significant difference between treatments and control at the level of P<0.05.

The germination of *B. campestris* and *P. minor* when grown in non-sterile or sterile soil was >90%. No significant difference in the germination of either species was observed. Root length of either *B. campestris* or *P. minor* seedlings was not suppressed in non-sterile soil treated with 80, 160 or 240 µg HQ/g non-sterile soil (Figure 1). A significant (P<0.05) reduction in the root length of both *B. campestris* (except 80 µg HQ/g sterile soil) and *P. minor*, however, was observed in sterile soil treated with different amounts of HQ (Figure 1).

**Figure 2:**
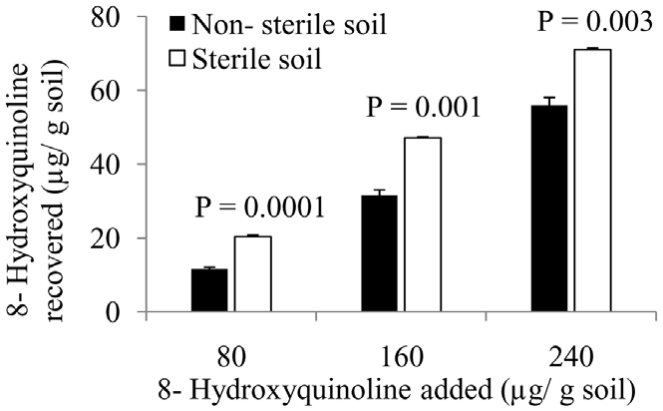
8-Hydroxyquinoline recovery from sterile or non-sterile soil treated with 80, 160 or 240 µg HQ/g soil. Bars indicate 1 SE.

The concentration-dependent recovery of HQ in both non-sterile and sterile soil was observed (Figure 2). The recovery of HQ was significantly higher in sterile soil treated with 80 (t = −12.635, df = 4, P_(2-tailed)_ = 0.0001), 160 (t = −10.290, df = 4, P_(2-tailed)_ = 0.001) or 240 (t = −6.747, df = 4, P_(2-tailed)_ = 0.003) compared to non-sterile soil treated with similar amounts of HQ (Figure 2).

**Figure 3:**
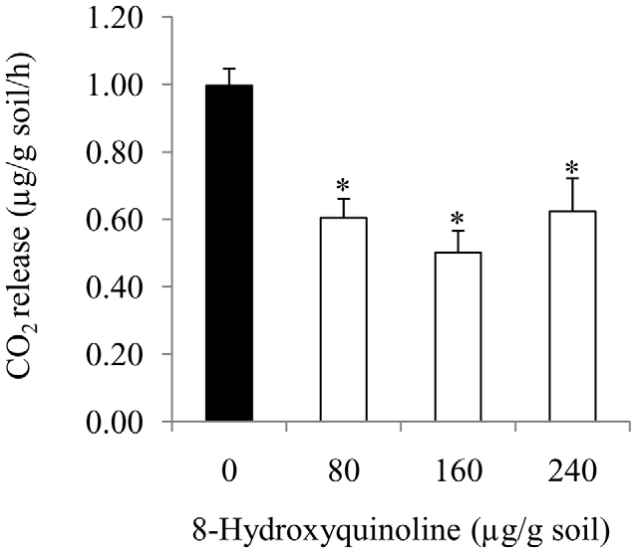
Microbial activity as determined by CO_2_ release (µg CO_2_ released/g soil/h) of soil treated with 0, 80, 160 or 240 µg 8-Hydroxyquinoline/g soil. Bars indicate 1 SE. Asterisks indicate significant difference between treatments and control (0 µg HQ/g soil) at the level of P<0.05.

Significantly lower CO_2_ release was observed in soil treated with 80 (t = 5.274, df = 8, P_(2-tailed)_ = 0.001), 160 (t = 3.383, df = 8, P_(2-tailed)_ = 0.010) or 240 (t = 6.111, df = 8, P_(2-tailed)_ = 0.000) µg HQ/g soil compared to untreated soil (Figure 3).

**Figure 4:**
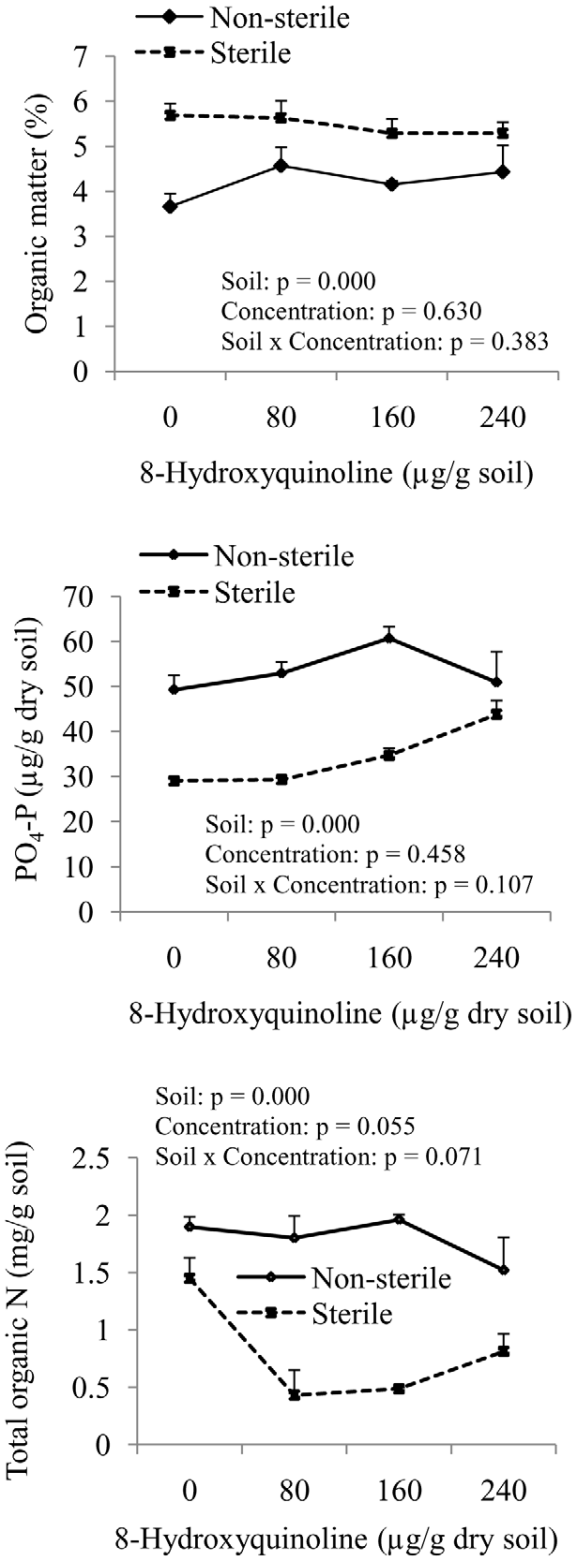
Soil organic matter (SOM), phosphate-P and total organic N levels of sterile or non-sterile soil treated with 0, 80, 160 or 240 µg 8-hydroxyquinoline/g soil. Bars indicate 1 SE. Two way ANOVA was carried out for the effect of soil sterilization and HQ concentration and their interaction on SOM, PO_4_-P and total organic N levels in soil.

Non-sterile soil treated with 80, 160, and 240 µg HQ/g soil did not have significant differences in SOM, PO_4_-P or total organic N compared to untreated control (Figure 4). Sterile soil had lower total organic N when treated with 80 (P = 0.009) or 160 (P = 0.013) µg HQ/g sterile soil. Exchangeable PO_4_-P in sterile soil treated with 240 µg HQ/g sterile soil significantly (P = 0.002) increased compared to untreated control (Figure 4). Soil organic matter of HQ treated sterile soils, however, was not different from untreated sterile soil. In order to check the effect of sterilization versus HQ treatment on SOM, PO_4_-P or total organic N, we performed two-way ANOVA. While soil sterilization had significant impact on the levels of organic matter (F = 26.521, P = 0.000), PO_4_-P (F = 44.431, P = 0.000) or total organic N (F = 46.638, P = 0.000), HQ did not influence of organic carbon (F = 0.591, P = 0.630), PO_4_-P (F = 0.911, P = 0.458) or total organic N (F = 3.130, P = 0.055). The interaction of soil sterilization and HQ concentrations in soil was not significant for organic carbon (F = 1.086, P = 0.383), PO_4_-P (F = 2.386, P = 0.107) or total organic N (F = 2.846, P = 0.071).

### Organic Matter Modification

**Figure 5:**
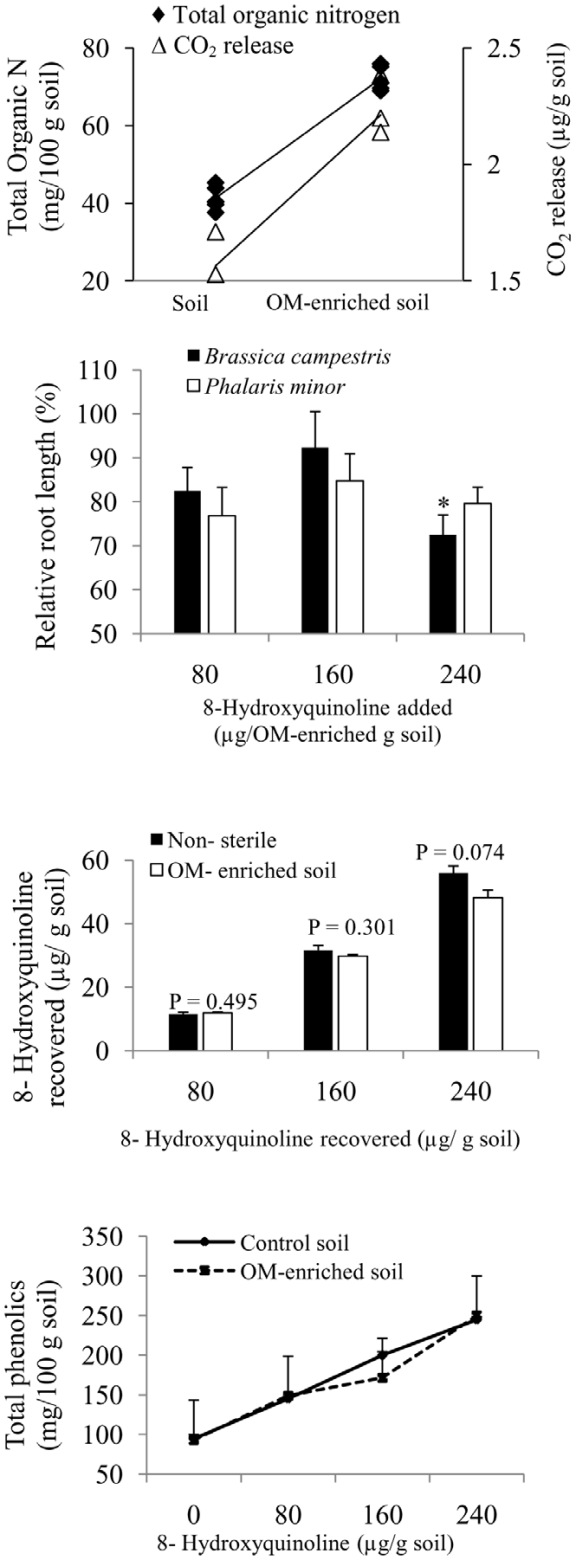
Organic matter (OM)-enriched soil had higher values of total organic nitrogen and CO_2_ release compared to soil not enriched with OM (control soil). We examined the impacts of HQ-amended OM-enriched soil on plant growth, HQ recovery and total phenolics content of soil. (A) Total organic N and CO_2_ release in soil enriched with organic matter and unamended control soil, (B) Relative root length (%) of *Brassica campestris* and *Phalaris minor* when grown in OM-enriched non-sterile soil treated with 0, 80, 160 or 240 µg 8-hydroxyquinoline/g soil. Root length of untreated control (0 µg HQ/g soil) is taken as 100%, (C) Recovery of 8-hydroxyquinoline from organic matter-enriched non-sterile soil treated with 0, 80, 160 or 240 µg HQ/g soil, (D) Total phenolic content of 8-hydroxyquinoline from OM-enriched non-sterile soil treated with 0, 80, 160 or 240 µg HQ/g soil. Bars indicate 1 SE. Asterisks indicate significant differences in the treatment from control at the level of P<0.001.

Organic matter-enriched soil had significantly higher total organic N (74.4%) and CO_2_ release (41%) compared to non-sterile soil not modified with OM (Figure 5A). Root length of *B. campestris* (except 240 µg HQ/g soil) or *P. minor* was not inhibited in OM-enriched soil treated with different amounts of HQ (Figure 5B). Recovery of HQ in OM-enriched soil treated with 80, 160 or 240 µg HQ/g soil was not different from unmodified non-sterile soil (Figure 5C). An increase in total phenolic content of both non-sterile and OM-enriched soil treated with HQ was observed (Figure 5D). Total phenolic content of both soils, however, was not different.

### Glucose Amendment

**Figure 6:**
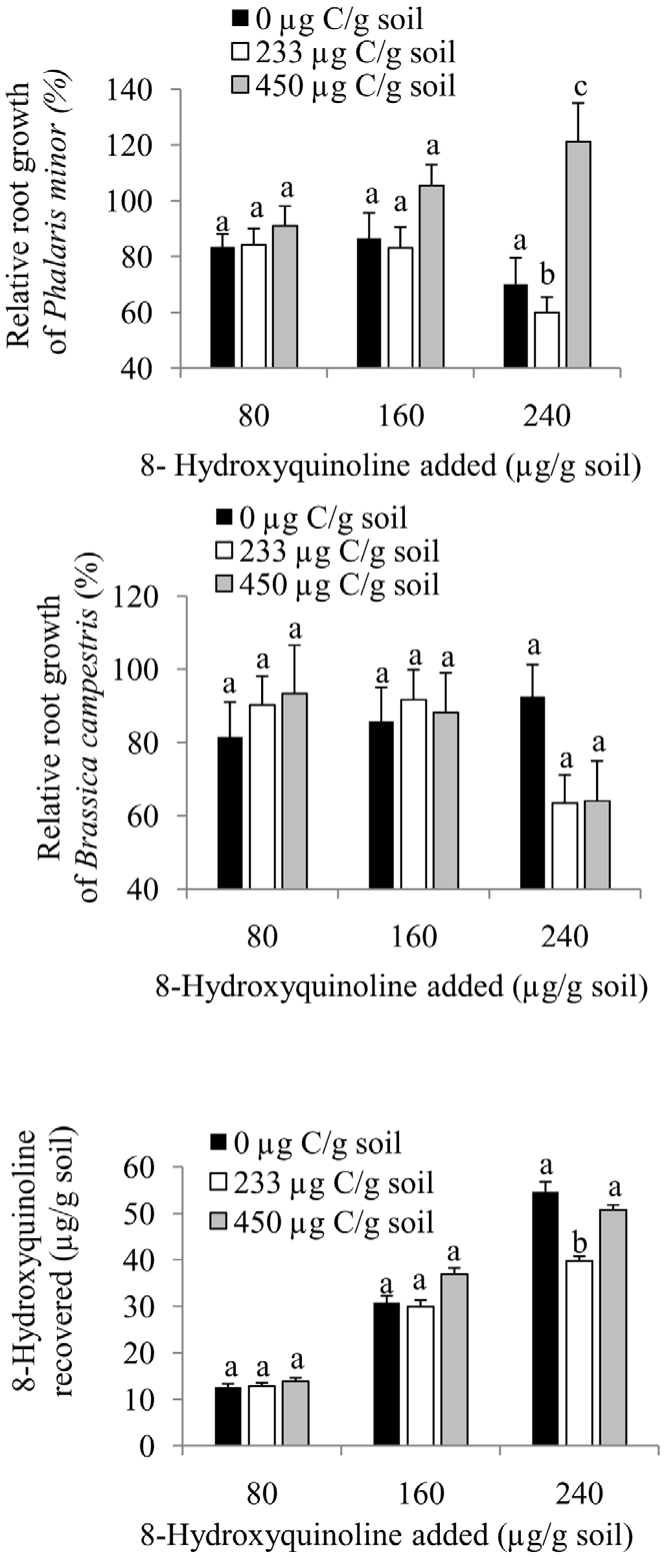
The impact of HQ-amended C-modified soil on root growth of *Phalaris minor* (A) and *Brassica campestris*, and HQ recovery was studied. (A and B) Relative root length (%) of *P. minor* (A) and *B. campestris* (B) when grown in C-amended (0, 233 or 450 µg C/g soil) soil treated with 0, 80, 160 or 240 µg 8-hydroxyquinoline/g soil. Root length of untreated control (0 µg HQ/g soil) is taken as 100%, and root length of both the assay species were calculated to the zero level of HQ, (C) Recovery of 8-hydroxyquinoline from C-amended (0, 233 or 450 µg C/g soil) soil treated with 80, 160 or 240 µg 8-hydroxyquinoline/g soil. Bars indicate 1 SE.

Glucose amendments (233 or 450 µg C/g soil) did not have any significant impact on the root growth of either *B. campestris* or *P. minor* (except when treated with 240 µg HQ/g soil) (Figure 6A, B). Root growth of *P. minor* was significantly suppressed in 233 µg C-amended soils treated with 240 µg HQ/g soil compared to untreated 233 µg C-amended soils (Figure 6A). An increase in the root length of *P. minor* was observed in 233 µg C-amended soils treated with 240 µg HQ/g soil compared to untreated 233 µg C-amended soils (Figure 6A). Although there was a concentration-dependent increase in HQ recovery in HQ-treated soil previously amended with 0, 233 or 450 µg C/g soil, there was no difference in the recovery of HQ among soils amended with 0, 233 or 450 µg C/g soil except a decrease in soil amended with 233 µg C/g soil when treated with 240 µg HQ/g soil (Figure 6C).

## Discussion

In non-sterile soil bioassays, HQ did not suppress the root growth of either *B. campestris* or *P. minor* but significant inhibition of root growth of both the assay species was observed in HQ-amended sterile soil (Figure 1). This could be explained due to higher recovery of HQ in sterile soil when treated with 80 (41.7%), 160 (36.5%) or 240 (39.5%) µg HQ/g soil compared to non-sterile soil treated with 80 (23.6%), 160 (24.5%) or 240 (31.1%) µg HQ/g (Figure 2). The observed higher recovery of HQ in sterile soil compared to non-sterile soil could be due to microbial or chemical transformation of HQ in non-sterile environment [19]–[24]. The primary metabolites of quinoline microbial transformations were reported to be 8-hydroxycoumarin, 2-hydroxyquinoline, 2,8-dihydroxyquinoline and 2,3-dihydroxyphenylpropionic acid [20], [25], [26]. The quality and quantity of primary metabolites of HQ, however, depends upon specific soil microbe, and pH and phosphate content of soil [27]. Phytotoxic activities of coumarin and its derivatives are known [28]–[30]. Although microbial and non-microbial transformation of soil allelochemicals plays an important role in determining allelopathic activities [31], allelopathic potential of primary metabolites of HQ largely not worked out. 8-Hydroxyquinoline, however, plays an important role as a chelating agent [2], [7], [32]. Iron-HQ complex also reported to suppress the chemical oxidation of pyrite (FeS_2_), a most abundant sulphide associated with coal mines [27]. Probably HQ is more important as a chelating agent than as an allelopathic compound.

Vivanco et al. [6] observed ∼90% mortality of North American plant species (such as *Festuca idahoensis, Koeleria micrantha* and *Agropyron repens*) after the addition of ∼80-µg HQ/g soil (the concentration of HQ reported in Eurasian *C. diffusa* soils) to rhizosphere of populations of North American natives. These authors argued that North American species were not adapted to HQ compared to Eurasian plant species. In the present study, similar concentrations of HQ, however, did not have any influence on either of the bioassay species. Neither soil nor assay species taken in the present study was adapted to HQ. The lack of any inhibition of bioassay species in HQ-treated non-sterile soil indicated that either HQ does not accumulate in soil at phytotoxic levels or that it chelates with metal ions to form non-toxic complex [7]. Our results were consistent with the observations of Norton *et al.* [2] that HQ does not exhibit any allelopathic effects. 8-hydroxyquinoline, however, might exert antimicrobial activities, which was evident from the lower microbial activity in the HQ-treated soils compared to untreated soil (Figure 3). Vivanco et al. [6] reported antibacterial activities of HQ against *Xanthomonas campestris, Pseudomonas syringae, Agrobacterium radiobacter, Erwinia carotovora* and *E. amylovora* ***.***

Although soil sterilization might help to some extent in assessing the role of microbes in allelopathy, it does not distinguish effects of secondary metabolites from that of resource competition. Soil sterilization has significant impact on organic matter, PO_4_-P and total organic N (Figure 4). Reduction in total organic N and PO_4_-P in the sterile soil was observed compared to non-sterile soil, which might have influenced the growth performance of assay species [33]. Root growth inhibition of *B. campestris* and/or *P. minor* in sterile soil could be due to higher recovery of HQ in sterile soil and/or lower availability of PO_4_-P and total organic N. There is, however, a need to examine the role of soil sterilization in allelopathy bioassays, a widely employed technique to evaluate the role microbes in deciding the fate of allelochemicals [34]. The effects of sterilization on the fate of allelochemicals in soil depend upon on chemical nature of the molecule. Weidenhamer and Romeo [31] found that arbutin, hydroquinone and gallic acid could remain stable in sterilized soil but benzoquinones break down relatively quickly in the sterilized soil. These findings on the discriminate respone of allelochemicals to soil sterilization are important in terms of our understanding the fate of allelochemicals in microbe-free soil environment. Besides influencing the fate of allelochemicals, soil sterilization influenced soil properties [35]. The observed growth inhibition of assay species in HQ-treated sterile soil could be due to high HQ recovery or lower nitrogen or phosphate-P levels or both compared to non-sterile soil. In order to evaluate the role of microbes in determining fate of chemicals in soil, sterilization of soil is often employed as a technique [34]. Sterilization in addition to killing soil microbes could also influence the availability of nutrient that may have an impact of growth of bioassay species. It therefore becomes difficult to isolate the effects of allelochemicals on plant growth from other factors such as soil nutrients. Any misinterpretation of the role of sterilization in assessing allelopathic effects may misconstrue allelopathic effects.

We indirectly checked the possibility of chemical oxidation of HQ by studying its recovery and phytotoxic activities in HQ-amended OM-enriched soil because OM might coat HQ and prevent it from metal oxidation [9]. We also quantified the total phenolic content in HQ-amended OM-enriched soil. In general, root growth of *B. campestris* (except 240 µg HQ/g soil) and *P. minor* was similar in unmodified and OM-enriched soil (Figure 5B), which is explained by non-significant differences in HQ recovery in two soils (Figure 5C). Inderjit et al. [36] did not find any inhibition of either shoot height or biomass of *Bambusa arundinacea* when grown in non-sterile soil treated with 266 or 400 µg catechin/g soil. There was, however, significant inhibition of shoot height and biomass of *B. arundinacea* when grown in OM-enriched soil treated with similar amounts of catechin. Lack of growth inhibition of assay species in HQ-treated OM-enriched soil further suggested that HQ may not be a potent allelopathic compound. The degradation of HQ in non-sterile soil is indirectly explained by an increase in total phenolics in HQ-treated non-sterile and OM-enriched soil (Figure 5D). The observed increase of total phenolic levels in HQ-treated soils (unmodified and OM-enriched) with increasing concentration of HQ treatment could be due to microbial degradation of HQ into simpler phenolic compounds [22], [25], but further experimental evidence is needed.

We thought that noninhibitory concentrations of glucose might influence HQ phytotoxicity. Compared to plant-released chemicals such as phenolics, glucose is considered as a preferred labile C source for microbes [10]. Pue et al. [11] found that noninhibitory concentrations of glucose increased the inhibitory activity of *p*-coumaric acid on *Ipomoea hederacea* biomass accumulation. In general, we did not observe any inhibition of root growth of either bioassay species when HQ-treated soils when amended with glucose. This suggests that alternate C-rich source does not reduce the microbial degradation of HQ.

Our data and previous work on HQ [2], [7] suggest that (i) HQ may not be a growth inhibitor at concentrations found in soil but may form complexes with trace elements and (ii) inhibit microbial activity. A plant-released chemical may not inhibit plant growth at a given concentration but may have important ecological role in terms of influencing soil microbial and chemical factors [5], [7], [37]. Instead of arguing allelopathic potential of an allelochemical only through plant growth bioassays, the ecological role(s) of the allelochemical should be examined in addition to its effect on plant growth. Further, efforts should be mode to design novel techniques to quantify allelochemicals in soil environment. Through solid-phase extraction, Erdem et al. [32] found that natural betonite could effectively adsorb HQ from aqueous solutions. The adsorption of HQ onto betonite, however, was pH dependent. Future studies on the adsorption and recovery of HQ from aqueous and soil solutions might help to further understand its role in the soil environment [38].

Our results imply that the phytotoxicity of an allelochemical depends upon the biological, chemical, and physical composition of the soil environment. Abiotic and biotic conditions of native soils might be different from those of non-native soils, which might play an important role in accumulation and phytotoxicity of an allelochemical. Second, this study adds to a growing literature demonstrating that extraction methodology and growth media are important factors in determining phytotoxicity of an allelochemical.
